# Protective effects of human iPS-derived retinal pigmented epithelial cells on retinal degenerative disease

**DOI:** 10.1186/s13287-020-01608-8

**Published:** 2020-03-04

**Authors:** Deliang Zhu, Mengyuan Xie, Fabian Gademann, Jixing Cao, Peiyuan Wang, Yonglong Guo, Lan Zhang, Ting Su, Jun Zhang, Jiansu Chen

**Affiliations:** 1 Key Laboratory of Optoelectronic Information and Sensing Technologies of Guangdong Higher Educational Institutes, Jinan University, Guangzhou, China; 2 Key Laboratory for Regenerative Medicine, Ministry of Education, Jinan University, Guangzhou, China; 3 Eye Institute, Medical College of Jinan University, Guangzhou, China; 4 Aier Eye Institute, Furong Middle Road, Changsha, China

**Keywords:** Retinitis pigmentosa, Retinal degeneration, hiPSC-RPE, rd10

## Abstract

**Background:**

Retinitis pigmentosa (RP) is an inherited retinal disease characterized by progressive loss of photoreceptor cells. This study aim at exploring the effect of retinal pigment epithelium (RPE) derived from human-induced pluripotent stem cell (hiPSC-RPE) on the retina of retinal degeneration 10 (rd10) mice, which are characterized with progressive photoreceptor death.

**Methods:**

We generated RPE from hiPSCs by sequential supplementation with retinal-inducing factors and RPE specification signaling factors. The three-dimensional (3D) spheroid culture method was used to obtain optimal injectable hiPSC-RPE cells. Subretinal space transplantation was conducted to deliver hiPSC-RPE cells into the retina of rd10 mice. Neurotrophic factor secretion from transplanted hiPSC-RPE cells was detected by enzyme-linked immunosorbent assay (ELISA). Immunostaining, Western blotting, electroretinography (ERG), and visual behavior testing were performed to determine the effects of hiPSC-RPE on the retinal visual function in rd10 mice.

**Results:**

Our data demonstrated that hiPSC-RPE cells exhibited classic RPE properties and phenotype after the sequential RPE induction from hiPSCs. hiPSC-RPE cells co-cultured with mouse retinal explants or retinal ganglion cells 5 (RGC5) exhibited decreased apoptosis. The viability and functional properties of hiPSC-RPE cells were enhanced by 3D spheroid culture. Transplanted hiPSC-derived RPE cells were identified by immunostaining with human nuclear antigen staining in the retina of rd10 14 days after subretinal space injection. The pigment epithelium-derived factor level was increased significantly. The expression of CD68, microglial activation marker, reduced after transplantation. The light avoidance behavior and ERG visual function in rd10 mice improved by the transplantation of hiPSC-RPE cells.

**Conclusion:**

Our findings suggest that injectable hiPSC-RPE cells after 3D spheroid culture can rescue the structure and function of photoreceptors by sub-retinal transplantation, which lay the foundation for future clinical cell therapy to treat RP and other retinal degeneration diseases.

## Background

Retinal degeneration is caused by genetic and environmental damage which leads to visual impairment. Retinitis pigmentosa (RP) is an irreversible and incurable inheritable retinal disease characterized by the progressive loss of photoreceptors and abnormalities in the retinal pigment epithelium. Initial symptoms are night blindness, a reduced visual field, and eventually complete blindness [1]. With a prevalence rate of approximately 1/4000, more than 2 million people are affected by RP [1–3]. Therefore, the development of novel therapies for RP is important. Stem cell-derived cell transplantation might be a promising treatment for RP, and clinical trials are going on.

A wide range of animal models was used to study the retinal degeneration. The rd1 mouse was the earliest animal model for RP. It carries a nonsense mutation in exon 7 of the beta subunit of cGMP-phosphodiesterase (PDE), which leads to the rapid and massive death of photoreceptors. Compared to rd1, the rd10 mouse bears a missive mutation in exon 13 of the PDE gene [4–7]. The rod photoreceptor cells of rd10 mice begin to degenerate at postnatal day 17 (P17) and attain a peak at P25. Majority of rod loss at P35 while complete loss is observed by P50 [8, 9]. With a later onset and slow retinal degeneration, rd10 mice are suitable to understand the progression of human RP. These properties make the rd10 mouse an eligible/adequate model to study the pathogenesis and investigate the treatment options for human RP. In this study, the rd10 mouse was used as a model for retinal degeneration to examine the mechanisms of cell death. Furthermore, RPE cells derived from induced pluripotent stem cells (iPSCs) were used for therapy to develop new therapeutic strategies.

Because of their pluripotent features, iPSCs and embryonic stem cells (ESCs) are considered to be the main source for regenerative medicine. iPSC cells bypass ethical issues and immunological challenges when compared to ESCs [10, 11]. Both iPSCs and ESCs are able to differentiate into retinal pigment epithelium (RPE) cells exhibiting many features of native RPE cells in vitro. Previous studies have reported that intact RPE is highly associated with photoreceptor survival [12]. The RPE monolayer resides between the neural retina and Bruch’s membrane. RPE cells play an important role in retinal homeostasis and photoreceptor maintenance. They perform several major functions in the eye, including light absorption to protect photoreceptors from photooxidation, transepithelial transport of nutrients, and ions; secretion of neurotrophic factors; phagocytosis of shedding photoreceptor outer segments; and maintenance of the visual cycle [13]. The pigment epithelium-derived factor (PEDF) is constantly secreted by the RPE cells. Many studies have suggested that PEDF could protect retinal cells from injury and death [12, 14–16]. Therefore, RPE cells might be promising for the treatment of retinal degeneration.

RPE cells were first successfully transplanted into the subretinal space of the Royal College of Surgeon (RCS) rats in 1988 [17]. Further studies on the treatment of retinal degeneration investigated various RPE cell types [5, 14, 18–20]. Nowadays, RPE cell transplantation is widely accepted as the treatment of retinal degeneration. Recently, in clinical trials (phase 1/2 studies), human ESC-derived RPE has been used to treat Stargardt’s macular degeneration and age-related macular disease (AMD) [21, 22]. da Cruz et al. [23] used hESC-derived RPE patches transplanted into wet AMD patients. Meanwhile, Takahashi’s group conducted the first human clinical trial of an autologous iPSC-RPE sheet to treat wet AMD patients [24]. These clinical studies demonstrated that RPE transplantation might be a relatively safe way and promising way to treat retinal degeneration. Based on these studies, we hypothesized that hiPSC-RPE transplantation could counteract the retinal degeneration in the rd10 model of RP. We tried to use injectable RPE cells after 3D spheroid culture for the subretinal space transplantation in rd10 mice model.

The objective of this study is to evaluate the protective effects of the hiPSC-RPE cells on rd10 mouse retinal degeneration in vitro and in vivo together with its molecular mechanisms. For this purpose, we generated RPE cells derived from hiPSCs. Co-culturing of hiPSC-RPE cells with either retinal explant or retinal ganglion cells 5 (RGC5) was conducted to explore the potential factors from hiPSC-RPE cells against retinal degeneration. In addition, hiPSC-RPE cells were transplanted into the subretinal space of rd10 mice, and the effect on the phenotype, function, visual behavior, and microglial activation was examined.

## Materials and methods

### Animals

C57BL/6 J and rd10 mice were originally purchased from Jackson Laboratory (Bar Harbor, USA). All experimental procedures of animals were approved by the Committee for Animal Care, Jinan University (Guangzhou, China), and all procedures pertaining to the use of animals were in accordance with the Association for Research in Vision and Ophthalmology (ARVO) statement. In all procedures, mice were weighed and anesthetized by intraperitoneal injection of 1% pentobarbital sodium (70 mg/kg body weight, Sigma-Aldrich, USA). Animals were maintained on a 12-h light-dark cycle and were housed in the Animal Laboratory of Jinan University. Age-matched littermates were used as controls. The mice were systemically immunosuppressed with cyclosporine A and prednisolone.

### Cell culture and differentiation

hiPSCs were purchased from Saibei company (Beijing, China). hiPSCs were cultured and passaged using mTeSR1 medium (BD Biosciences) on Matrigel-coated plates. The procedure of hiPSCs differentiation into RPE was modified as previously described [25]. Briefly, hiPSCs were cultured in basal medium containing Dulbecco’s modified Eagle’s medium/F12 (DMEM/F12) (Gibco, Grand Island, NY), 1× B27 supplement (Invitrogen), 1× N2 supplement (Invitrogen), 1× nonessential amino acids (Gibco, NY) supplemented with 50 ng/mL Noggin (R&D systems, Minneapolis, MN) and 10 ng/mL human recombinant insulin-like growth factor-1 (IGF1) (R&D Systems), and 10 mM nicotinamide (R&D systems) for 2 days to initiate differentiation. On the third day, cells were cultured in the presence of basal medium supplemented with 10 ng/mL (Noggin, Dkk1, IGF1; R&D systems) and 5 ng/mL basic fibroblast growth factor (bFGF; R&D systems) and 10 mM nicotinamide (R&D systems) for 2 days. On the fifth day, the cells were cultured in base medium supplement with 10 ng/mL DKK1 (R&D systems), 10 ng/mL IGF1, and 100 ng/mL Activin A (R&D Systems) for 2 days. From days 6 to 14, cells were cultured in the presence of basal medium containing 100 ng/mL Activin A (R&D Systems), 10 M SU5402 (Millipore, Germany), and 1 mM VIP (Sigma-Aldrich). Lastly, enriched RPE-like cells were cultured in DMEM-HG supplement with 1% fetal bovine serum (FBS), 100 ng/mL Activin A, sodium pyruvate, and Gluta MAX (Invitrogen) for 10 days. All hiPSC-RPE cells were used in this study starting with the first passage.

### Immunocytochemistry and immunohistochemistry

Immunostaining was carried out to confirm cell-specific and tissue-specific markers. For this purpose, the cultured cells or cryostat sections were washed with PBS and fixed with 4% paraformaldehyde for 15 min and then washed twice with PBS. Then, they were incubated in PBS-3% bovine serum albumin (BSA)-0.3% Triton X-100 at room temperature for 1 h followed by incubation with primary antibodies (rabbit polyclonal anti-OCT4, 1:400, CST; mouse polyclonal anti-Sox2, 1:200, Abcam; rabbit monoclonal anti-ZO-1, 1:400, Abcam; mouse polyclonal anti-Mitf, 1:400, Santa Cruz; rabbit anti-human nuclear antigen, Abcam; mouse anti-CD68, Abcam; rabbit polyclonal anti-Brn3b, 1:200, Proteintech; rabbit polyclonal anti-RPE65, Abcam) overnight at 4 °C. Following this, the cells or cryostat sections were incubated with goat anti-mouse IgG H&L Alexa Fluor®488/goat anti-rabbit IgG H&L Alexa Fluor®555 (1:500, Abcam) for 2 h at room temperature. The cell nucleus was stained with 4′,6-diamidino-2-phenylindole (DAPI) for 5 min at room temperature. Images were captured with fluorescence microscopy (Observer 7, Zeiss).

### Co-culture system

The co-culture system platform is shown in Fig. 2a. Retinal explant was co-cultured with hiPSC-RPE to study the effect on retinal cells. Firstly, the eyes were enucleated, and the cornea, lens, and vitreous body were removed. Subsequently, retinal explants were gently detached from the choroid pigment layer. Retinal explants of about 10 mm^2^ were divided into four equal sections and mounted on the bottom of the six-well transwell (Millipore, USA). The cells were seeded out with a concentration of 2 × 10^5^ cells on the top membrane of the insert (pore size 0.22 μm; Millipore, USA). Cultivation was conducted at 37 °C in 5% CO_2_ for 2 days. The co-cultured system cells were cultured in DMEM/F12 containing 10% FBS, 100 IU/mL penicillin, and 100 μg/mL streptomycin (P/S). Retinal explants cultured without hiPSC-RPE cells were used as the control group. Finally, the apoptosis of retinal cells on retinal explants was detected by terminal deoxynucleotidyl transferase dUTP nick end labeling (TUNEL) staining.

RGC5 cell line originated from rat RGC, which express Brn3b and other markers specific for RGC [26, 27]. The RGC5 (Shanghai cell bank of Chinese Academy Sciences) and hiPSC-RPE cells were grown in the co-culture system for 48 h, which allowed for the diffusion of soluble factors (Fig. 2c). RGC5 cultured without hiPSC-RPE cells were used as the control group. RGC5 apoptosis and death were detected by flow cytometry (BD Biosciences).

### Flow cytometry

Flow cytometric analysis using annexin V-FITC and propidium iodide was used to detect apoptosis and cell death. For this purpose, the cells were digested using 0.25% trypsin, washed twice with PBS, and incubated with annexin V-FITC and propidium iodide for 30 min. The experiments were performed with triplicates for each group. Following this, apoptosis and cell death were detected by the flow cytometer (BD Biosciences). BD FACSDiva 8.0.1 was used for the analysis of flow cytometric data.

### TUNEL assay

A commercial terminal TUNEL (Roche, Germany) was purchased to examine the apoptosis. The assay was performed according to the manufacturer’s instructions. Images were captured using fluorescence microscopy (Observer 7, Zeiss).

### 3D spheroid culture

3D Petri dish micromolds (Merck Micro Tissue, USA) were used to culture spheroid hiPS-RPE cells as previously described [28]. Briefly, the rubber templates were sterilized with 70% ethanol and ultraviolet irradiated for 2 h. Autoclaved agarose was pipetted in templates to imprint microwells on agarose hydrogel. After solidification, the microwell agarose mold was removed on a six-well plate. Two hundred microliters of cell suspension solutions was pipetted (approximately 1 × 10^5^ cells) onto agarose microwell molds. Cells seeded onto micro-molds of agarose settle into the small recesses and self-assembled into spheroids.

### Viability assay

Calcein AM and EthD-III double staining (Molecular Probes, USA) were carried out according to the manufacturer’s instructions. The spheroid hiPSC-RPE cells dissociated into single-cell seed on a six-well plate cultured for a day. Cultured cells were stained using 2 μM Calcein-AM and 4 μM EthD-III for live and dead cell staining, respectively. Calcein-AM displays a green signal indicating living cells, and EthD-III displays a red signal indicating dead cells. The images of staining were observed by fluorescence microscopy (Olympus, Japan).

### Transepithelial electrical resistance assay

A transepithelial electrical resistance (TEER) assay was used to assess the dynamic barrier function of the epithelioid cells [29]. Cells were seeded into 24-transwell inserts at 1 × 10^4^ cells/ insert. The dynamic barrier of the cells was determined through measuring TEER across the cell monolayer using Millicell-ERS-2 (Millipore, Temecula, USA). The value of TEER was calculated according to the following equation:

TEER (Ω cm^2^) = (*R*_total_ − *R*_insert_)/A

*R*_total_ is the resistance measured (Ω), *R*_insert_ (Ω) is the resistance of the insert *w*, and *A* is the membrane area (cm^2^) of the insert.

### Subretinal space transplantation

Transplantation surgical procedures were performed on mice at P12 (a few days earlier before the onset of photoreceptor degeneration). Animals were anesthetized with an intraperitoneal injection of pentobarbital sodium, and the pupils were dilated with tropicamide. The mice were placed on a heating pad at 37 °C and operated under direct visual control using a stereomicroscope (Leica, Germany). A trans-scleral incision was made with a 31-gauge syringe (BD) in the mice eye. One microliter of hiPSC-RPE cells (approximately 2 × 10^5^ live cells per microliter) was delivered into the subretinal space through a small scleral incision made by a syringe with a 33-gauge needle (Hamilton, Switzerland). After the hiPSC-RPE cells were injected, a small bleb appeared in the retina. The needle was used to handle this bleb for a few seconds to prevent the reflux of the same in the vitreous humor. During the same procedure, WT mice received sham surgery.

### Enzyme-linked immunosorbent assay

The functionality of hiPSC-RPE in the rd10 retina was further evaluated by their PEDF secretion. For this purpose, PEDF concentration was detected by the enzyme-linked immunosorbent assay (ELISA) kit (Biovendor, Karasek, Czech Republic). Retina tissue extract fluid specimens were collected from hiPSC-RPE-transplanted-rd10, nongrafted-rd10, and WT at P26. The ELISA was carried out according to the manufacturer’s instructions and analyzed using a microplate photometer.

### Histological assessment

Morphological analyses were performed at retinal sections in the region of the mid-peripheral retina at 1 mm radial distance from the optic nerve. rd10 mice and age-matched WT were analyzed from P17, P26, P45, P60, and P180, to characterize retinal degeneration over time. Mouse eyes were fixed in 4% paraformaldehyde (PFA) at room temperature for 15 min. The cornea and lens were removed in PBS, and eyecups were consecutively incubated in 10%, 20%, and 30% sucrose until the tissue sunk to the bottom of the plate. Thereafter, the tissue was embedded in paraffin/tissue cryoprotective medium. For further use, 5-μm-thick sections were cut dorso-ventrally through the optic nerve head, air-dried, and stored at 80 °C. Retinal sections on slides were de-waxed with xylene and ethanol, washed with PBS, and then stained with hematoxylin and eosin or DAPI. Images were captured using the microscope.

### Western blots

Proteins of mouse retina tissue were extracted with a protein extraction solution assay kit (RAPI, Sigma). Nanodrop (Thermo) was used to measure protein concentration. Equal amounts of proteins were separated on 10% sodium dodecyl sulfate-polyacrylamide gel electrophoresis (SDS-PAGE) resolving gel and 5% stacking gel and transferred onto polyvinylidene fluoride (PVDF) membranes. PVDF membranes were blocked with skim milk (BD) in Tris-buffered saline (TBS) for 1 h at room temperature. Following this, they were incubated with primary antibodies at 4 °C overnight (rabbit monoclonal anti-ZO-1, 1:500, Abcam; mouse monoclonal anti-RPE65, 1:500 Abcam; mouse polyclonal anti-Nestin, 1:500 Santa Cruz; rabbit monoclonal anti-α-SMA, 1:1000, Abcam; mouse polyclonal anti-CD68, 1:1000, Proteintech; rabbit anti-Bax, 1:1000, Abcam; rabbit monoclonal anti-GAPDH, 1:1000, Abcam). This was followed by PVDF membranes being washed with TBS-T (Tween 1:1000 dilution in TBS buffer) three times followed by incubation with secondary antibodies (HRP-goat anti-rabbit IgG, 1:2000, Bioss; HRP-goat anti-mouse IgG, 1:2000, Bioss) for 1 h at room temperature. After incubation, PVDF membranes were washed three times with PBS. Protein signals were detected with SuperSignal™ West Femto Maximum sensitivity substrate (Thermo Fisher Scientific) and imaged using the chemiluminescence system (Bio-Rad).

### Light avoidance behavior testing

Mouse visual behavior was tested in a platform with a partitioned arena equal-sized (25 cm, 25 cm, and 25 cm) dark and light chambers connected by an aperture through which the mouse was allowed to transition freely. The mouse was placed in a dark room before the test. The light chamber was set up, and an LED array was suspended above the chamber emitting dim green light. The chambers were thoroughly cleaned with 70% ethanol before each test. The pupils were dilated using tropicamide 10 min prior to the test. The mice were released in the middle of the lit chamber facing away from the connecting aperture. Light avoidance was estimated by the percentage of time spent in the dark chamber. Data were recorded by a digital camera mounted above the lit chamber and calculated by ANY-Maze video tracking software.

### Electroretinography

Electroretinography was applied to detect the retinal function of hiPSC-RPE-transplanted rd10 mice and age-matched non-transplanted rd10 and WT mice. After overnight dark adaption, mice were anesthetized with an intraperitoneal injection of pentobarbital sodium, and pupils were dilated with 1% tropicamide. The corneal surface was anesthetized with 0.5% proparacaine. During the electroretinography (ERG) test, the mice were placed on a platform to maintain body temperature at 37 °C. For flash ERG recordings, two gold wire loop corneal electrodes were attached to the eyes. Additionally, two reference electrodes were attached to the forehead, and a ground electrode was attached to the tail. ERG data was recorded with a RETI-scan system (Roland Consult, RETI-scan, Germany). ERG responses measured were performed under two conditions: scotopic/dark-adapted (light intensities, 0.1, 0.3, 1, 3, 10 cd-s/m^2^) and photopic/light-adapted (light intensities, 1, 3, 10 cd-s/m^2^). The photopic detection was conducted after the mice were exposed to background light of 30 cd-s/m^2^ intensity for 10 min. Analysis of a-wave and b-wave amplitudes was performed using ERG data analyzer software.

### Statistical analyses

SPSS software (version 21.0; IBMSPSS Inc., Chicago, IL, USA) and GraphPad PRISM (version 5.0; GraphPad Inc., La Jolla, CA, USA) were used for statistical analysis and to create graphics. The summarized data were expressed as mean ± SEM obtained from at least three independent experiments. For multiple comparison among groups, one-way analysis of variance (ANOVA) followed by Tukey multiple comparison tests (equal variances) or Dunnett’s T3 multiple comparison tests (unequal variances) was performed. For comparison between two groups, the unpaired two-tailed Student’s *t* test was performed. Statistical significance was defined as *p* < 0.05. The following convention was used to indicate *p* values: “NS” indicates *p* ≥ 0.05, one asterisk indicates 0.01 ≤ *p* < 0.05, and two asterisks indicate *p* < 0.01.

## Results

### Characterization of hiPSCs and hiPSCs-RPE

**Fig. 1:**
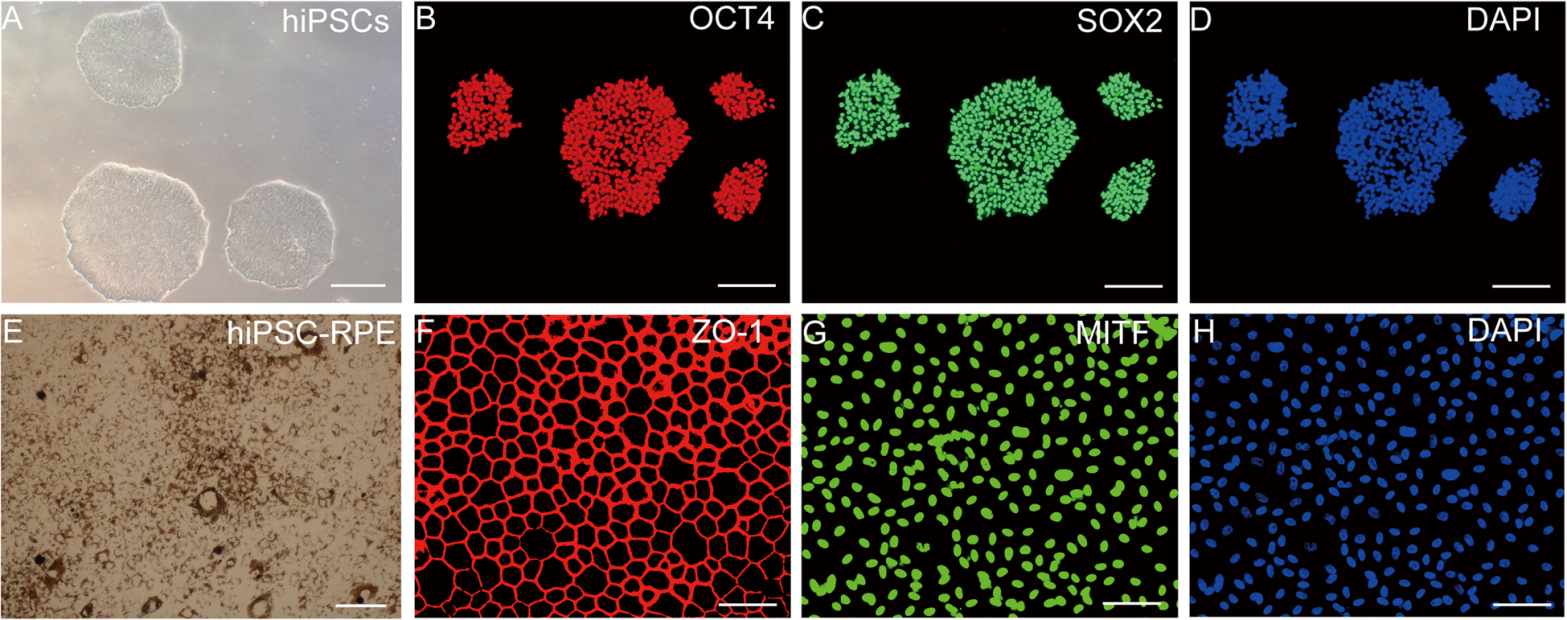
Characterization of hiPSCs and hiPSC-RPE. **a** Brightfield images of hiPSCs exhibiting typical colonies morphology. **b**, **c** hiPSCs expression of pluripotency markers OCT4 and SOX2. **d**, **h** Cell nucleus staining with DAPI. **e** Brightfield images of hiPSC-RPE cells display melanin pigmentation and polygonal morphology. **f**, **g** hiPSCs-RPE expression of typical RPE markers, cell boundaries when stained with ZO-1, and nuclear staining with MITF. Scale bar 200 μm (**a**, **e**) and 100 μm (**b**, **c**, **d**, **f**, **g**, **h**)

hiPSCs were used to generate RPE cells (Additional file 1: Figure S1). hiPSCs displayed typical clonal morphology, and their pluripotency was verified by traditional pluripotent stem cell markers OCT4 and SOX2 (Fig. 1a–d). Differentiation of hiPSCs into RPE cells was achieved by exposure to different growth and signaling factors [25]. hiPSCs-derived RPE showed pigmentation and polygonal morphology (Fig. 1e). Intrinsic RPE markers were used to validate the expression of hiPSC-RPE, including zonula occludens (ZO-1) tight junction proteins and microphthalmia-associated transcription factor (MITF) (Fig. 1f–h). These results indicate an efficient differentiation of hiPSCs into RPE cells.

### Anti-apoptosis effect of hiPSC-RPE on the retinal cells in vitro

**Fig. 2:**
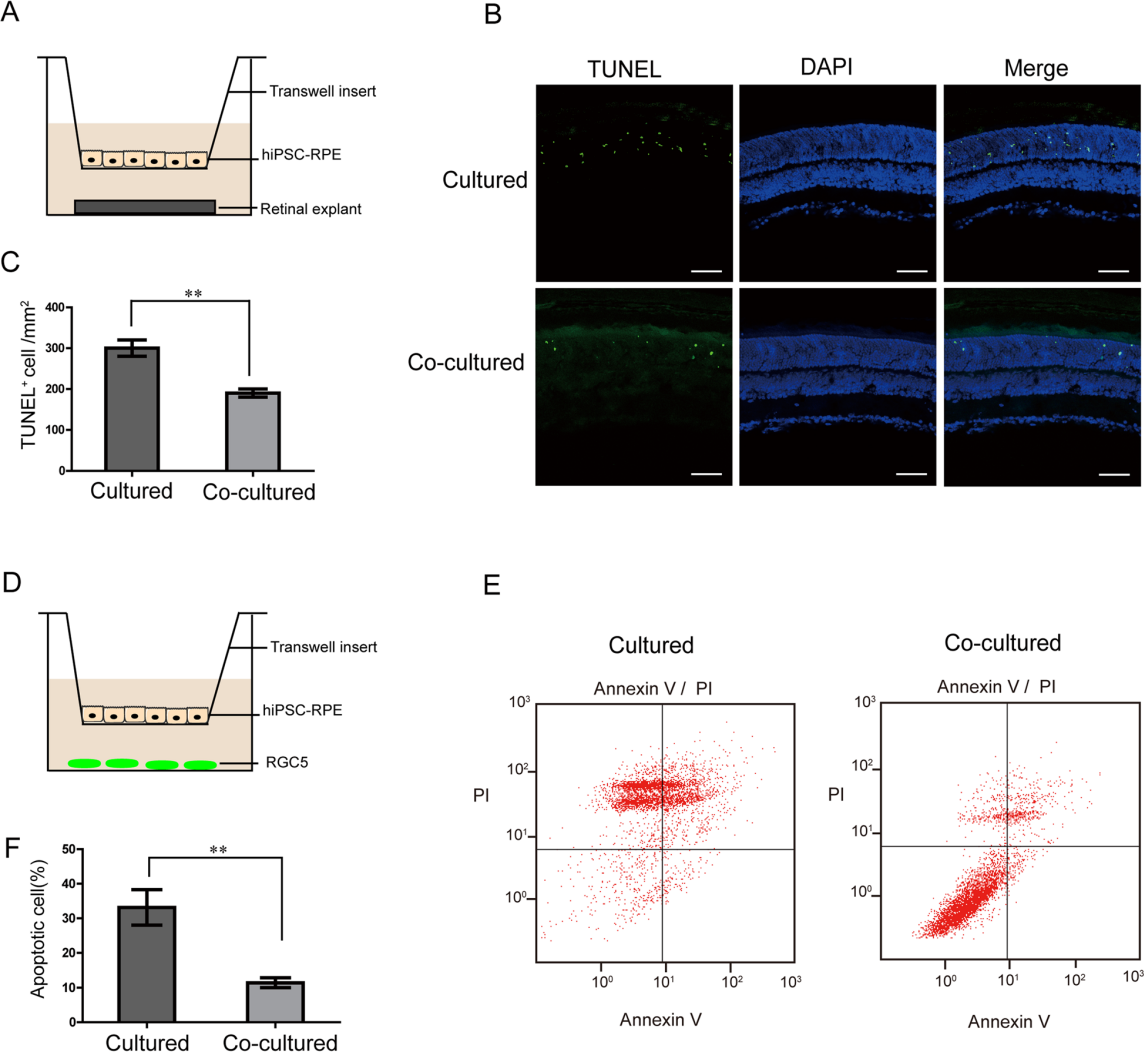
Protective effect of co-culturing of retinal explants and RGC5 with hiPSC-RPE in vitro. **a** Model of co-culture system: hiPSC-RPE cells co-cultured with retinal explants separated by transwell insert. **b** TUNEL/DAPI co-staining of the retinal explant after co-culturing with and without hiPSC-RPE cells after 2 days. **c** Bar chart representing the positive TUNEL staining of cells per square millimeter compared between retinal explants with or without co-culturing. **d** Illustration of the transwell co-culture system: RGC5 co-cultured with hiPSC-RPE cells. **e** Flow cytometry analysis of apoptosis in RGC5 co-cultured with and without hiPSC-RPE. **f** Bar chart shows the rate of apoptosis in percentage compared between RGC5 with and without co-culturing. Scale bar 50 μm (**b**). Data are presented as mean ± SEM. *p* values were determined by unpaired two-tailed Student’s *t* test (**c**, **f**), *n* = 4 for each group, *p < 0.05, **p < 0.01

A co-culture system was used to analyze the effect of hiPSC-RPE cells on retinal explants and RGC5 (Fig. 2a, d). RGC5 cell line showed neural shape and positive Brn3b staining (Additional file 2: Figure S2). TUNEL staining demonstrated that co-culturing with hiPSC-RPE significantly decreased the apoptosis of retinal explants when compared to non-co-cultured control group (Fig. 2b, c). In addition, flow cytometry analysis revealed that the ratio, including early and late apoptotic, of RGC5 apoptosis was remarkably reduced by co-culture with hiPSC-RPE cells (Fig. 2e, f). These findings revealed a protective effect of iPSC-derived RPE cells on retinal cells by reducing apoptosis and cell death.

### Promotion of hiPSC-RPE viability and maintenance of properties and functions by spheroid culture

**Fig. 3:**
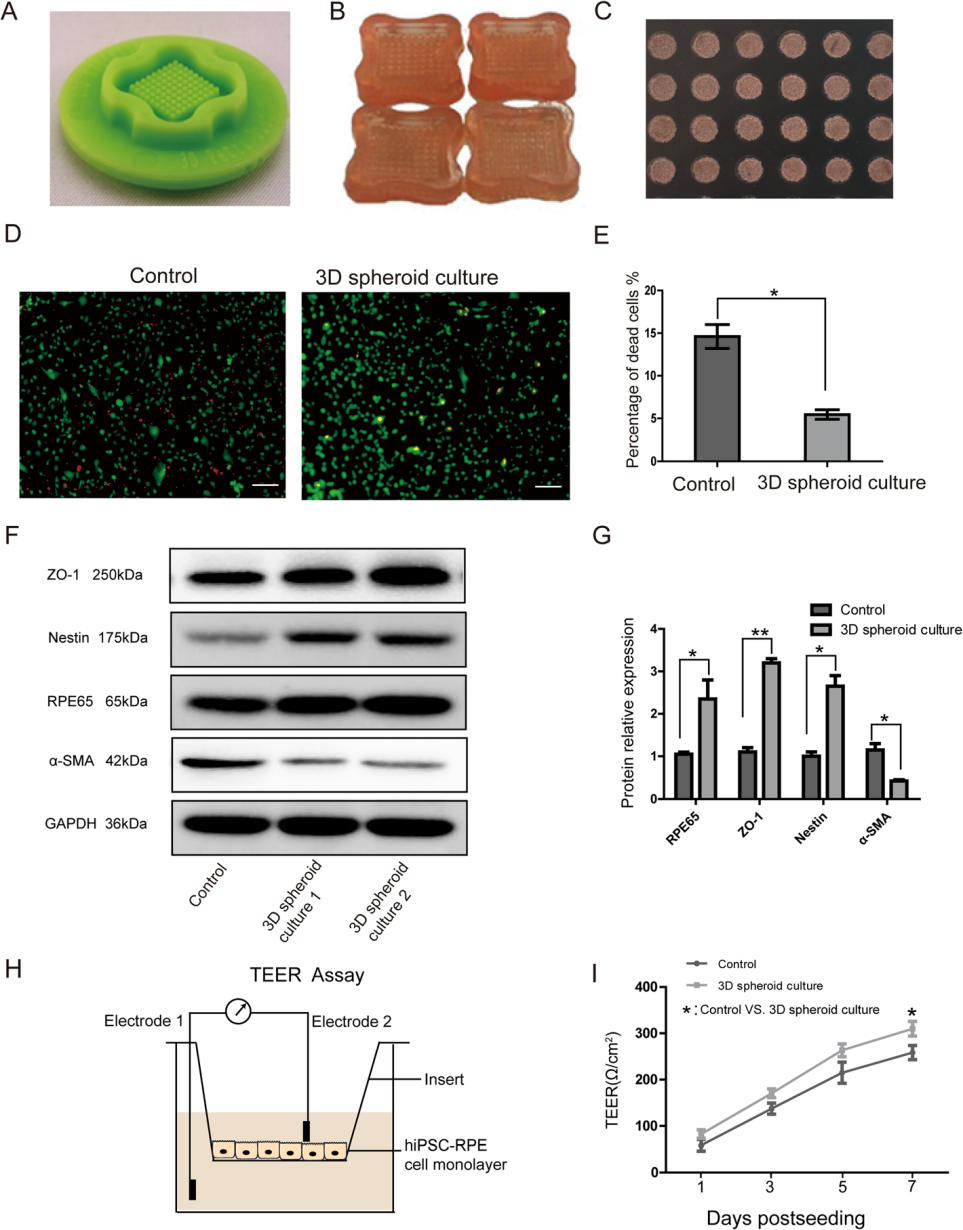
3D spheroid culture improved the viability of hiPSC-RPE and helped in maintaining RPE properties and functions. **a** A rubber micro-mold with multiple projections. **b** Agarose mold with imprint microwells. **c** Formation of hiPSC-RPE cell spheroid in agarose 3D Petri dishes. **d** Viability staining of dispersed hiPSC-RPE cell spheroid. Non-spheroid cultured hiPSCs were used as control. **e** Bar chart represents the percentage of dead cells of the control compared to 3D spheroid cultured hiPSC. **f** Immunoblotting analysis: the protein expression of ZO-1, RPE65, Nestin, and α-SMA in hiPSC-RPE after spheroid culturing. **g** Bar chart representing relative protein expression of several RPE-specific markers of 3D spheroid cultured hiPSC-RPE. **h** Schematic of TEER value measurements with the insert culture. **i** TEER assay results reveal that 3D spheroid culture could improve the hiPSC-RPE cells barrier function. Scale bar 200 μm (**d**). Data are presented as mean ± SEM. *p* values were determined by unpaired two-tailed Student’s *t* test (**f**), *n* = 5 for each group, *p < 0.05, **p < 0.01

To achieve a perfect condition of hiPSC-RPE cells for retinal degeneration therapy, microwell agarose mold was applied for 3D spheroid cell culture (Fig. 3a, b). hiPSC-RPE cells formed solid spheroids in agarose microwell mold after being cultured for 3 days (Fig. 3c). The viability of hiPSC-RPE cells was increased by 3D spheroid culturing (Fig. 3d, e). Also, the expression of intrinsic RPE-specific markers was increased, and α-SMA (a marker of myofibrosis) was decreased by spheroid culture (Fig. 3f, g). Moreover, hiPSC-RPE barrier functions were enhanced by spheroid culturing (Fig. 3h, i). Thus, 3D spheroid culture may be able to improve the viability and functional properties of hiPSC-RPE cells.

### hiPSC-RPE survival and PEDF secretion in the retina of rd10 mice

**Fig. 4:**
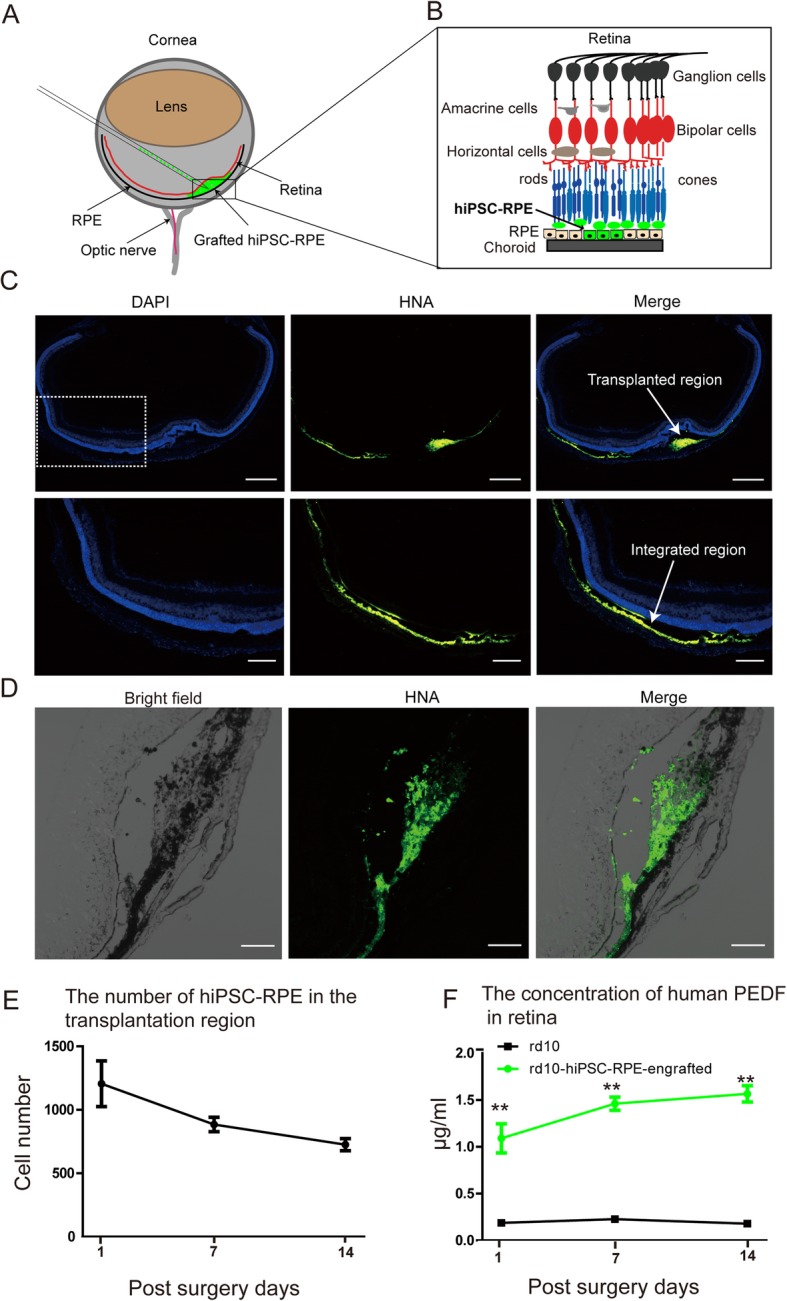
Survival of hiPSC-RPE and secretion of neurotrophic factors after transplantation into the rd10 mouse retina. **a** Schematic representation of hiPSC-RPE cell transplantation process into the subretinal space of rd10 mice. **b** Illustration of the hiPSC-RPE cells delivered into the potential space between retinal pigment epithelium and photoreceptors in the outer nuclear layer. **c** hiPSC-RPE cell survival analysis by HNA detected in transplanted rd10 retinal space day 7 post-surgery. **d** High magnification showed hiPSC-RPE cells were able to survive and integrate into host rd10 mouse retina by day 14 post-surgery. **e** The transplanted number of cells was 1000 ± 200, 800 ±100, and 700 ±100, at days 1, 7, and 14 post-surgery, respectively. **f** ELISA to quantify the concentration of PEDF in rd10 mice with and without transplantation. Scale bar 200 μm (**c**) and 50 μm (**d**). Data are presented as mean ± SEM. *p* values were determined by unpaired two-tailed Student’s *t* test (**f**), *n* = 4 for each group, *p < 0.05, **p < 0.01

hiPSC-RPE cells were injected into the subretinal space of rd10 mice at P12 (Additional file 3: Figure S3). The transplantation procedure is shown in Fig. 4a, b and Additional file 4: Figure S4. The formation of a bleb at the site of injection suggested a successful transplantation of donor cells. Transplanted cells were identified by immunostaining with human-specific antibodies against a human nuclear antigen (HAN) at days 5 (Fig. 4c) and 14 (Fig. 4d) post-surgery. Only a few hiPSC-RPE cells integrated into the host retina and transplant decreased with time (Fig. 4e). Moreover, the transplanted cells maintained the expression of RPE marker RPE65 (Additional file 5: Figure S5). The secretion of human PEDF by hiPSC-RPE after transplantation was detected by an ELISA. Compared with WT and non-transplanted cell models, the PEDF level significantly increased in rd10 mice that received hiPSC-RPE transplantation (Fig. 4f). These findings indicate that hiPSC-RPE cells could not only promote survival after injection but also secrete PEDF neurotrophic factor.

### Preservation of retinal structure and delay of retinal degeneration in rd10 mice by hiPSC-RPE transplantation

**Fig. 5:**
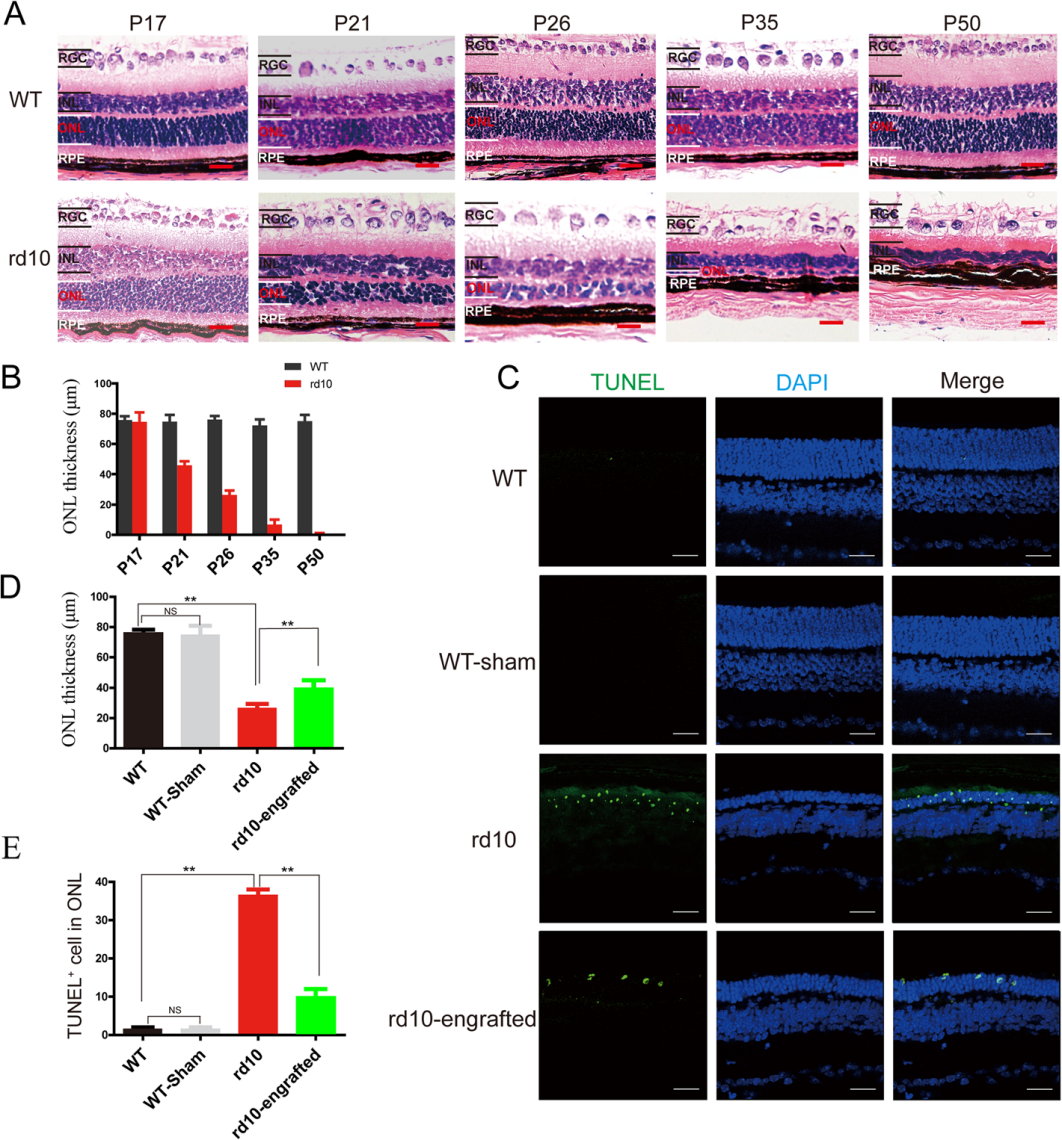
hiPSC-RPE cells preserved of photoreceptor in rd10 mice. **a** Histology of rd10 and WT mouse retina over time. **b** Quantification of the retinal outer nuclear layer thickness in rd10 and WT. **c** Retinal outer nuclear layer thickness and apoptotic cells in the retina of hiPSC-RPE-transplanted rd10 detected by DAPI and TUNEL staining, retinas from non-transplantation rd10 mice, and WT at the same age are used as a comparison. **d** Quantification of TUNEL-positive cell density. **e** Analysis of retinal outer nuclear layer thickness of after hiPSC-RPE cell transplantation. Scale bar 20 μm (**a**), 50 μm (**c**). Data are presented as mean ± SEM. *p* values were determined by one-way ANOVA followed by Tukey multiple comparison tests (**d**, **e**), *n* = 5 for each group, *p < 0.05, **p < 0.01

The thickness of the retinal outer nuclear layer (ONL) was reduced dramatically over time (Fig. 5a). Roughly 50% of the photoreceptors were reduced from P17 to P26, only two columns of photoreceptors remained at P45, and they were completely lost at P180. No distinct changes were observed to occur in WT mouse retinas with age (Fig. 5b). At P26 after hiPSC-RPE injection, the ONL thickness of rd10 mouse retina was greater than that in non-transplanted rd10 mice but a bit still thinner than that in WT mice (Fig. 5c, d). Furthermore, some apoptotic cells were found in WT mouse retina, whereas a large number of apoptotic cells were detected with TUNEL staining in rd10 mice. However, the number decreased in the retina of rd10 mice after the transplantation of hiPSC-RPE cells (Fig. 5e).

These results indicate that hiPSC-derived RPE cells might preserve the structure of the retina and attenuate photoreceptor loss in retinas of rd10 mice. Therefore, retinal degeneration in rd10 mice might be delayed by hiPSC-RPE cell transplantation.

### Inhibition of microglial activation by hiPSC-RPE transplantation

**Fig. 6:**
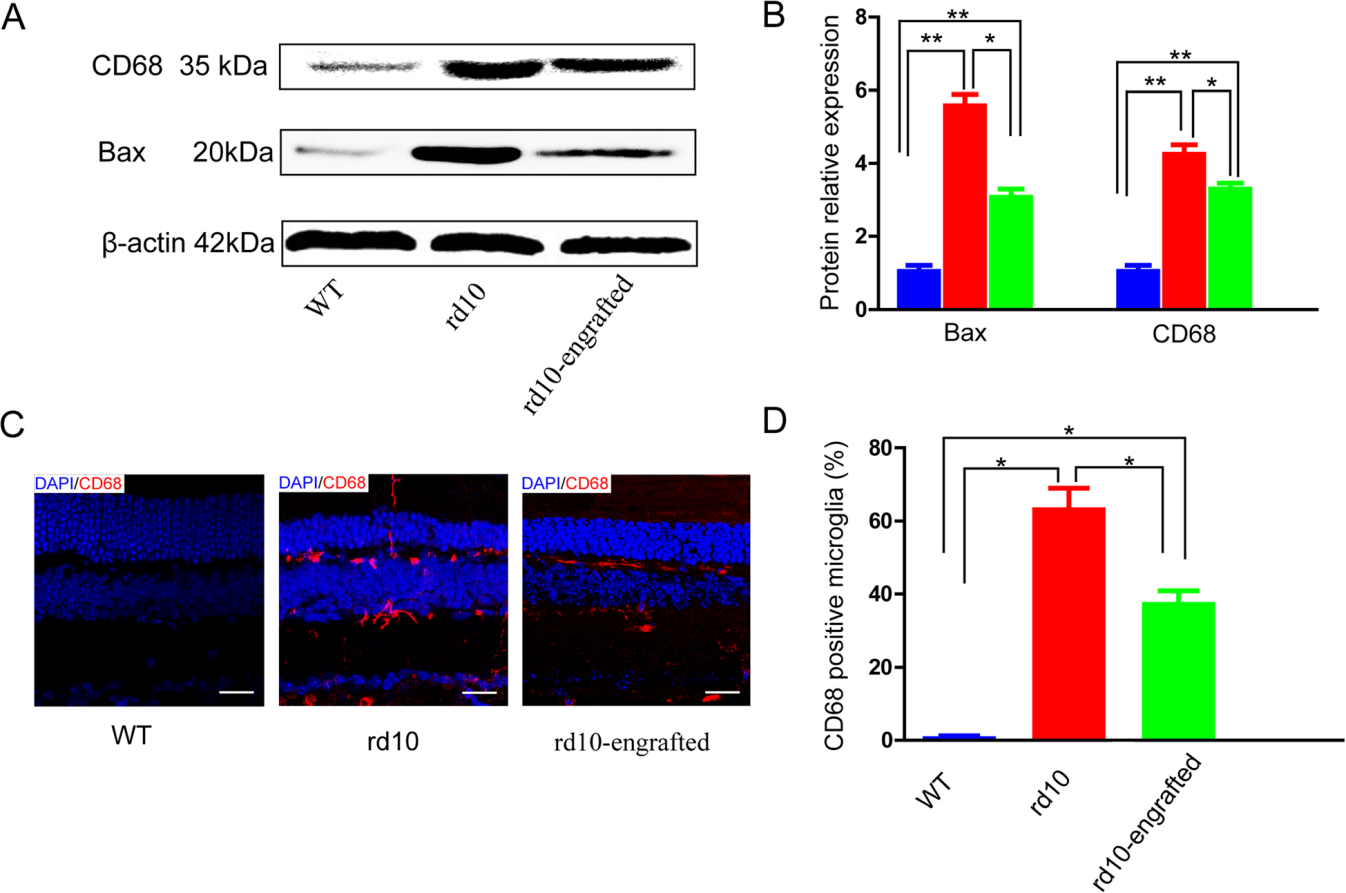
hiPSC-PRE suppressed the microglial activation and ameliorated the ocular environment condition in rd10. **a** Representative images of Western blot for CD68 and Bax. **b** Average protein expression of CD68 and Bax in rd10 mouse retina after hiPSC-RPE cell transplantation, retinas from non-transplantation rd10 mice, and WT at the same age are used for comparison. **c** Representative images of retinal immunohistochemistry for CD68. **d** Quantification of CD68-positive cell density. Scale bar 50 μm (**c**). Data are presented as mean ± SEM. *p* values were determined by one-way ANOVA followed by Tukey multiple comparison tests (**b**, **d**), *n* = 4 for each group, *p < 0.05, **p < 0.01

Microglia infiltration and activation are considered as causes of a degenerative retina [30]. Therefore, the effect of hiPSC-RPE on microglia activation in rd10 mouse retina was studied. Expression of CD68 (a marker of activated microglia) and Bax (a marker of pro-apoptosis) was observed in the retina of hiPSC-RPE-transplanted rd10 mice by Western blotting at 10 days post-surgery. Compared with WT, CD68 and Bax expressions were higher in rd10 mice, while both CD68 and Bax expressions were significantly decreased by hiPSC-RPE transplantation (Fig. 6a, b). In addition, immunostaining showed that CD68 was rarely present in the retina of WT mice. CD68 immunolabeling was stronger in the retinas of rd10 compared with those of hiPSC-RPE-transplanted rd10 mice (Fig. 6c, d). Taken together, our findings indicate that hiPSC-RPE cell transplantations suppress the microglial activation and ameliorate the pro-apoptosis conditions in rd10 degenerative retina.

### Improvement of visual behavior in rd10 mice by hiPSC-RPE transplantation

**Fig. 7:**
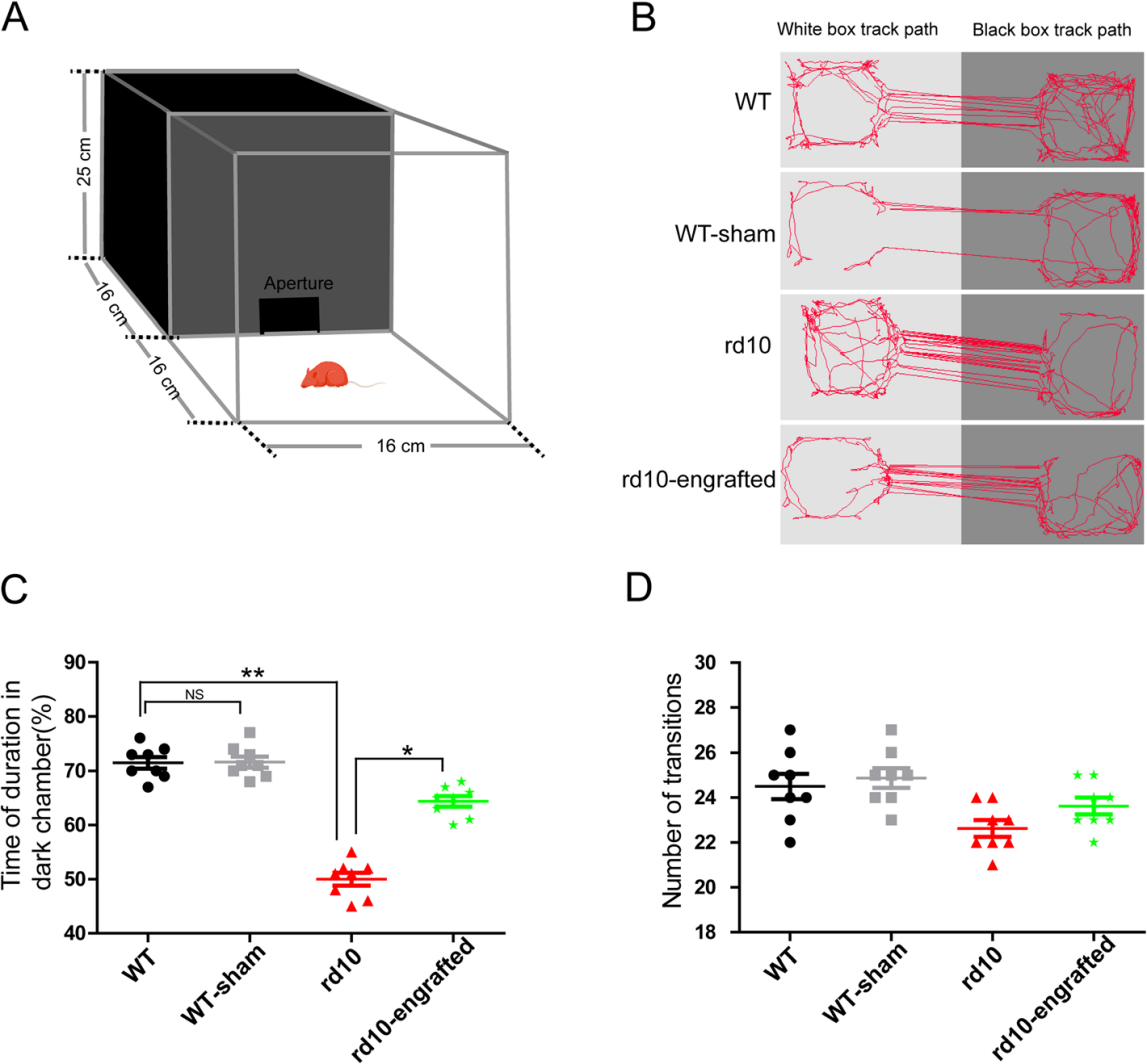
hiPSC-RPE improved rd10 mouse visual behavior. **a** Schematic representation of light-mediated behavior apparatus. **b** Track paths (red lines) of mice in black/white boxes. **c** Analysis of time duration spent by the mice in the dark compartment. **d** There are no distinct difference in the number of transitions between compartments among the groups. Data are presented as mean ± SEM. *p* values were determined by one-way ANOVA followed by Tukey multiple comparison tests (**c**, **d**). *n* = 8 mice per group, *p < 0.05, **p < 0.01

Light-mediated behavior apparatus was used to validate the photoreceptor function in mice (Fig. 7a). The results demonstrated that WT mice moved around the edge of the light chamber which they found aversive, while rd10 mice moved randomly in the light chamber, maybe due to bad vision. After the transplantation of hiPSC-RPE cells, rd10 mice showed improved visual behavior (Fig. 7b). The WT mice spent approximately 70% of the tested time in the dark chamber, whereas rd10 mice spent approximately 50% test time in the dark chamber. hiPSC-RPE-transplanted rd10 mice spent approximately 66% test time in the dark chamber. Compared with rd10, rd10 mice that received hiPSC-RPE transplants spent more time in the dark chamber (Fig. 7c). Furthermore, no difference was detected in the number of transitions between compartments among groups (Fig. 7d). Our findings suggest that hiPSC-RPE cells improve visual mediation behavior in rd10 mice.

### Improvement of visual function in rd10 mice by hiPSC-RPE transplantation

**Fig. 8:**
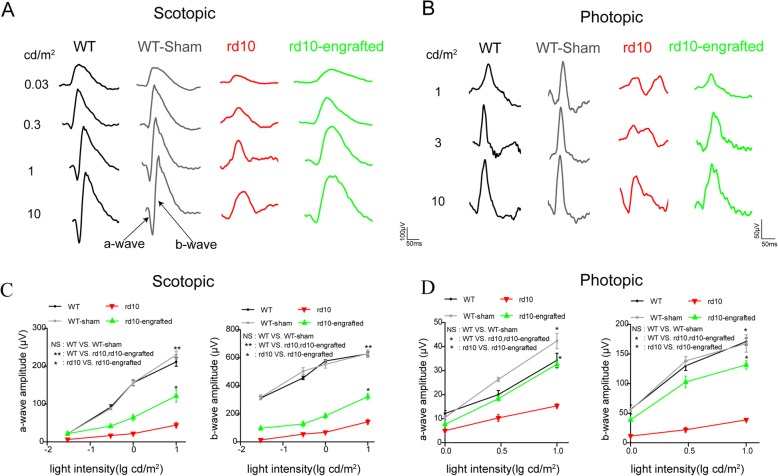
hiPSC-RPE preserved the photoreceptor function in rd10 mice. **a**, **b** Representative typical dark-adapted and light dark-adapted ERG response from rd10 at P26 after hiPSC-REP transplantation. Non-transplantation rd10 mice, WT, and WT-sham at the same age are used as a comparison. **c** Average scotopic a- and b-wave amplitudes elicited at 0.03, 0.3, 1, and 10 cd-s/m^2^ light intensities. **d** Average photopic a-wave and b-wave amplitudes elicited at 1, 3, and 10 cd-s/m^2^ light intensities. Results show that both under dark-adapted and light dark-adapted conditions, the ERG a-wave and b-wave amplitudes were significantly higher in the hiPSC-RPE-treated group than rd10 without transplantation. WT and WT-sham (injected with PBS) group ERG responses were not significant. Data are presented as mean ± SEM. *p* values were determined by one-way ANOVA followed by Dunnett’s T3 multiple comparison tests (**c**, **d**). *n* = 6 for each group. *p < 0.05, **p < 0.01

The visual function of rd10 mice was analyzed by ERG after cell transplantation. ERG response was examined for both dark-adapted and light-adapted conditions. ERG response was recorded under dark-adapted and light-adapted conditions. Under scotopic (Fig. 8a) and photopic conditions (Fig. 8b), both a-wave and b-wave amplitudes increased by light intensities in mice. At the same light intensity, both a-wave and b-wave amplitudes in rd10 mice were remarkably reduced compared with WT mice. Under both dark-adapted and light-adapted conditions, the ERG a-wave and b-wave amplitudes were significantly higher in the hiPSC-RPE transplantation rd10 group than the non-grafted rd10 group at P26 (Fig. 8c, d). For WT sham-operated eyes, no significant difference was observed compared with WT normal eyes. Thus, the results indicate that hiPSC-RPE cell transplantation might improve the ERG visual function in rd10 mice.

## Discussion

RP is an irreversible and incurable degenerative retinal disease, and its pathogenesis is poorly understood. In the present study, iPSCs-derived RPE cell transplantation was examined as a strategy for RP therapy. Our results demonstrated that iPSCs have the capability to differentiate effectively into RPE cells in vitro. The iPSC-derived RPR cells possess native RPE morphology and markers. hiPSC-RPE cells co-cultured with either retinal explants, or RGC5 could prevent retinal cells from entering apoptosis. In addition, optimal injectable hiPSC-RPE cells were obtained by 3D cell spheroid culturing. The results showed that hiPSC-RPE cells were able to improve the photoreceptors’ structure, function, and visual behavior in rd10 mice. Moreover, microglial activation was suppressed, and the retinal condition was ameliorated after hiPSC-RPE cells transplantation. In summary, our findings suggest that hiPSC-RPE cells might attenuate retinal degeneration.

Accumulating studies revealed that ESCs and iPSCs could be successfully differentiated into RPE cells in vitro [20, 25, 31–33]. The properties of RPE cells derived from ESCs or iPSCs were similar to classic RPE cells characterized by pigmentation, polarization, tight junction, and phagocytosis photoreceptor outer segment [34]. In our experiments, retinal-inducing factors, such as IGF-1, DKK1, Noggin, and bFGF, and RPE-specific signaling factors, such as nicotinamide, Activin A, and VIP, were used to generate RPE from iPSCs. hiPSC-RPE not only resembles native RPE morphology but also expresses RPE-specific markers.

To obtain the optimal injectable hiPSC-RPE cells, 3D spheroid culturing was used. The findings showed that 3D spheroid culture could improve the viability of hiPSC-RPE cells, maintain cell properties, and enhance the expression of intrinsic RPE-specific markers. 3D spheroid culture methods are known to improve cell-cell and cell-extracellular matrix interactions and thereby better resemble the in vivo environment [35, 36]. The viability and neural differential potential of adipose-derived stem cells (ADSC) enhanced by spheroid culturing have been shown previously [28]. To the best of the knowledge, this is the first time hiPSC-RPE cells were used after spheroid culturing for transplantation into the rd10 mice to model retinal degeneration. The 3D culture systems might provide an opportunity to enhance the efficacy of therapy to treat retinal degeneration.

In this study, no evidence of rejection or tumorigenesis after subretinal injection of hiPSC-RPE and transplanted cells could survive well for at least 2 weeks in the retina of rd10 mice. The hiPSC-RPE cell transplanted in rd10 retina space did not trigger immune response because the retina is an immune-privileged organ, and immunosuppressed drugs of cyclosporine A and prednisolone were administered post-transplantation. Xian and Huang reported that immunosuppressants are effective for the prevention of immune rejection of grafts within the subretinal space [37]. Sharma et al. demonstrated that at least 70% subretinal transplantation of hiPSC-RPE cells survives over a 10-week period. This was achieved by suppressing the systemic and resident innate immune responses using prednisone, doxycycline, and minocycline and the adaptive immune responses using tacrolimus and sirolimus [38]. Furthermore, a high concentration of human PEDF was detected in the retinal tissue extract fluid of rd10 transplanted with hiPSC-RPE. Retinal cells are vulnerable to various adverse factors such as inherited gene mutation, light glutamate, hydrogen peroxide, and ischemic injury. RPE cells secrete a series of neurotrophic growth factors beneficial for retinal cells and retinal homeostasis, including PEDF [39] and bFGF [40]. Various studies have shown that these factors can protect retinal cells from damage and death. Recently, researchers provided evidence demonstrating that the PEDF promoted photoreceptor survival in rd10 retina models.

Photoreceptor cells died rapidly in rd10 mice, because of rod photoreceptors carrying a mutation in PED6-beta. The mutation was associated with ER stress and oxidative stress which leads to reduction in photoreceptors [41]. hiPSC-RPE transplantation rescuing the overlying photoreceptors was observed, as suggested by increased thickness and a higher number of cells in the ONL in the transplanted area as compared to non-transplanted controls. Moreover, transplantation of hiPSC-RPE cells also improved the light-avoidance behavioral and visual function of rd10 mice. The findings are consistent with the results of the study by Tsai and colleagues, which showed that subretinal injection of neural progenitor cells (NPCs) derived from iPSCs preserved both photoreceptors and visual function [42]. Two main mechanisms are available for hiPSC-RPE cells to rescue retina from damage and death. The first one is part-transplanted hiPSC-RRE integration and replacement in the retina of rd10 mice, and the other one is the secretion of neuroprotective factors by the transplanted hiPSC-RPE cells as discussed above. This retinal neuroprotective effect by hiPSC-RPE cell secretion was also confirmed by our co-culture testing. Because our indirect co-culture system physically separates the hiPSC-RPE cells and retinal explants or RGC5, the diffusible factors released from hiPSC-RPE cells plated on transwell inserts confer neuroprotective effect. The neurotrophic factors released from hiPSC-RPE cells support retinal cell survival. So, the retinal explants or RGC5 can prevent retinal cells from degeneration after co-culture with hiPSC-RPE cells. Tanzina et al. also reported that adult porcine retinal explants co-cultured with human neural progenitor cells can slow down the degeneration process [43].

Microglia are resident macrophage cells of the retina. They play a vital role in clearing apoptotic cells. Retinal degeneration induces the microglial activation [30, 44, 45]. CD68 is frequently used as a marker for reactive microglia in response to retinal degeneration. In our study, the levels of CD68 were strongly increased in rd10 retina compared with the WT retina. Interestingly, the levels of CD68 were decreased in rd10 mice after hiPSC-RPE transplantation. Thus, hiPSC-RPE cells are involved in modulating the microglial activation and photoreceptor degeneration. The result was consistent with the study by Li et al. wherein the activation of microglia was suppressed by neural stem cells transplanted into rd1 subretinal space [46].

There are some limitations in this study. For example, the observation period in 2 weeks postoperative is rather short. Therefore, in the future, we will prolong the follow-up time after transplantation. Moreover, future development should also consider adding a control group with blank solution treatment, which can make the conclusion more convincible [47].

## Conclusion

In conclusion, this study demonstrated that iPSCs can differentiate into RPE cells when exposed to sequential supplementation with retinal-inducing factors and RPE-specific signaling factors. The viability of the hiPSC-RPE cell was enhanced/optimized by conducted 3D spheroid culture. 3D spheroid culture also helped in maintaining the hiPSC-RPE properties and functions. In vitro, hiPSC-RPE cells prevented retinal cells from apoptosis by indirect co-culture. In vivo, hiPSC-RPE cells were able to survive in rd10 mice up to 2 weeks post-surgery. hiPSC-RPE cells rescued and improved the reduced ONL, photoreceptor loss, impaired light-avoidance behavior, and ERG visual function, which are related to the mechanisms of PEDF neuroprotective factor release, inhibited microglia activation, part integration, and decreased cell apoptosis after subretinal injection of hiPSC-RPE cells. Our findings suggested that hiPSC-RPE cell transplantation is a promising strategy to preserve the structural and functional features of photoreceptors. Subretinal hiPSC-RPE cell transplantation is a potential therapy for retinal degeneration, and our method paves the way for further research and human trials. Still, many challenges remain to be solved to implement stem cell-derived cell therapy for degenerative retinal diseases. Future investigations should include longer observation time after transplantation and further track transplanted cell integration. The long-term safety and efficacy for hiPSC-RPE cells in rd10 mouse retina need to be determined.

## Supplementary information

**Additional file 1: Fig. S1** hiPSC-RPE differentiation protocol.

**Additional file 2: Fig. S2** Characterization of RGC5. a-c The morphology of RGC5 cell line. d-f RGC-specific marker Brn3b expression in RGC5. Scale bar 200 μm (a), 100 μm (b, d, e, f) and 50 μm (c).

**Additional file 3: Fig. S3** Flow chart of the experimental protocol of the animal study.

**Additional file 4: Fig. S4** Steps of subretinal transplantation in mice. a Dilated pupil before transplantation. b Cells delivery by a 33-gauge needle. **c** Blebs appeared in the retina after transplantation, the needle bypasses the lens, and the iris to reach the subretinal space followed by injection with 1 μl hiPSC-RPE cells.

**Additional file 5: Fig. S5** hiPSC-RPE cells maintained RPE cell marker RPE65 following subretinal transplantation of rd10. a Histological staining with RPE65 (green) and human nuclear marker (HNA) (red) of cross-sections were obtained from eyes at 2 weeks post-injection of hiPSC-RPE. b Transplanted hiPSC-RPE cells were co-stained with RPE65 (green) and HNA (red), DAPI (blue) stained nuclei. Scale bar 200 μm (a) and 100 μm (b).

## Data Availability

The datasets used and/or analyzed during the current study are available.

## Abbreviations

iPSC: Induced pluripotent stem cell
RPE: Retinal pigment epithelium
RP: Retinitis pigmentosa
3D: Three dimensional
ELISA: Enzyme-linked immunosorbent assay
DAPI: 4′,6-Diamidino-2-phenylindole
ERG: Electroretinography
RGC: Retinal ganglion cell
PEDF: Pigmented epithelium-derived factor
ESC: Embryonic stem cells
AMD: Age-related macular disease
IGF1: Insulin-like growth factor-1
bFGF: Basic fibroblast growth factor
FBS: Fetal bovine serum
PFA: Paraformaldehyde
ONL: Outer nuclear layer
P: Postnatal
RCS: Royal College of Surgeon
TEER: Transepithelial electrical resistance
HAN: Human nuclear antigen
