# Correction: Protective effects of human iPS-derived retinal pigmented epithelial cells on retinal degenerative disease

**DOI:** 10.1186/s13287-022-03097-3

**Published:** 2022-09-30

**Authors:** Deliang Zhu, Mengyuan Xie, Fabian Gademann, Jixing Cao, Peiyuan Wang, Yonglong Guo, Lan Zhang, Ting Su, Jun Zhang, Jiansu Chen

**Affiliations:** 1 Key Laboratory of Optoelectronic Information and Sensing Technologies of Guangdong Higher Educational Institutes, Jinan University, Guangzhou, China; 2 Key Laboratory for Regenerative Medicine, Ministry of Education, Jinan University, Guangzhou, China; 3 Eye Institute, Medical College of Jinan University, Guangzhou, China; 4 Aier Eye Institute, Furong Middle Road, Changsha, China

## Correction to: Stem Cell Research & Therapy (2020) 11:98 https://doi.org/10.1186/s13287-020-01608-8

Following the publication of the original article [1], the authors identified an error in the layout of the images in Fig. 5C, that the positions of WT DAPI stain and WT-sham DAPI stain images need to be swapped. The correct figure has been included in this correction. This error does not affect the conclusion of the paper.

**Fig. 5:**
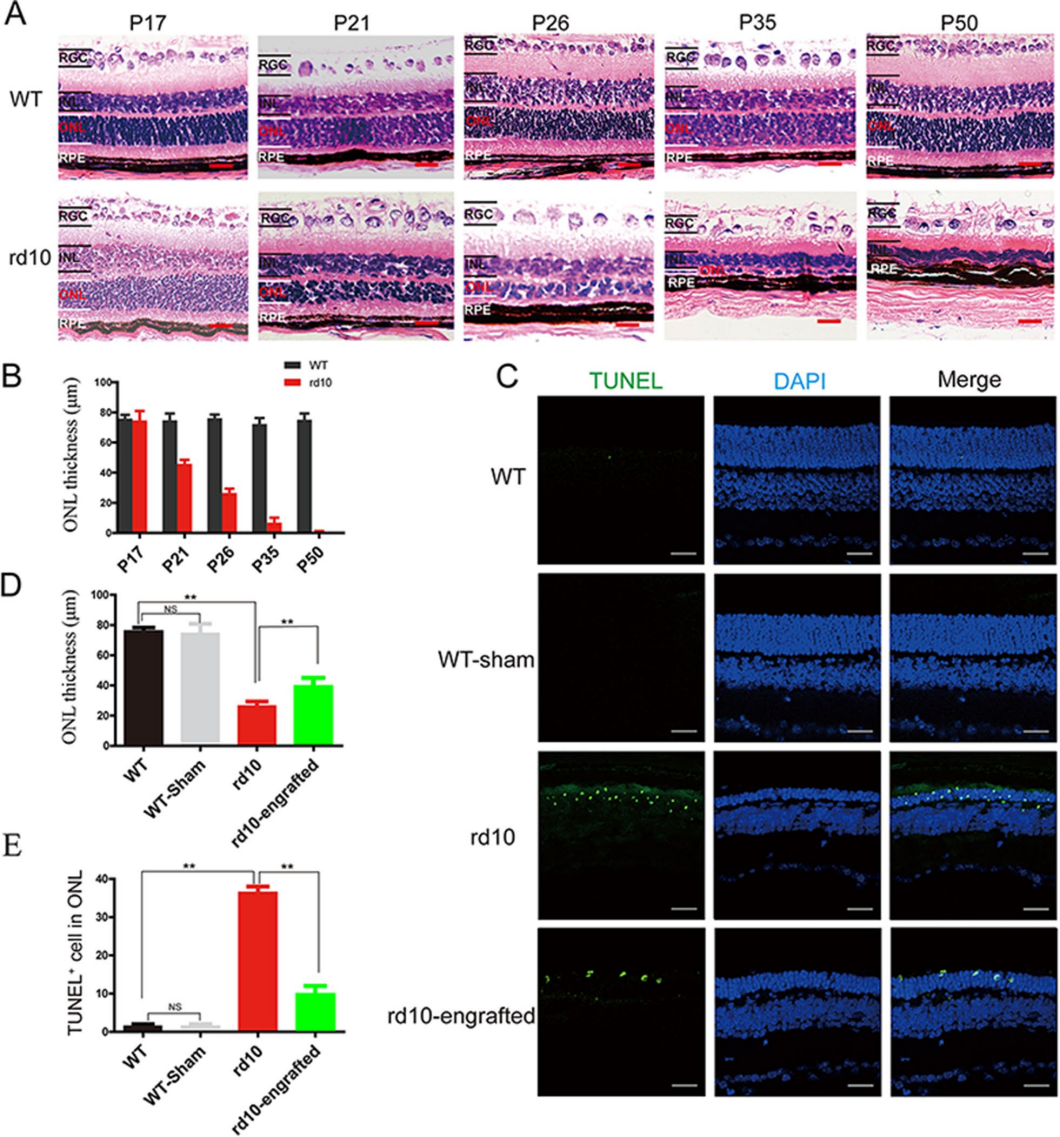
hiPSC-RPE cells preserved of photoreceptor in rd10 mice. **a** Histology of rd10 and WT mouse retina over time. **b** Quantification of the retinal outer nuclear layer thickness in rd10 and WT. **c** Retinal outer nuclear layer thickness and apoptotic cells in the retina of hiPSC-RPE transplanted rd10 detected by DAPI and TUNEL staining, retinas from non-transplantation rd10 mice, and WT at the same age are used as a comparison. **d** Quantification of TUNEL-positive cell density. **e** Analysis of retinal outer nuclear layer thickness of after hiPSC-RPE cell transplantation. Scale bar 20 μm (**a**), 50 μm (**c**). Data are presented as mean ± SEM. *p* values were determined by one-way ANOVA followed by Tukey multiple comparison tests (**d**, **e**), n = 5 for each group, *p < 0.05, **p < 0.01
