# Supplementary figures and images for: Protective effects of human iPS-derived retinal pigmented epithelial cells on retinal degenerative disease
Source: Stem Cell Res Ther. 2020 Mar 4;11:98. doi: 10.1186/s13287-020-01608-8 (PMC7055119; doi:10.1186/s13287-020-01608-8)

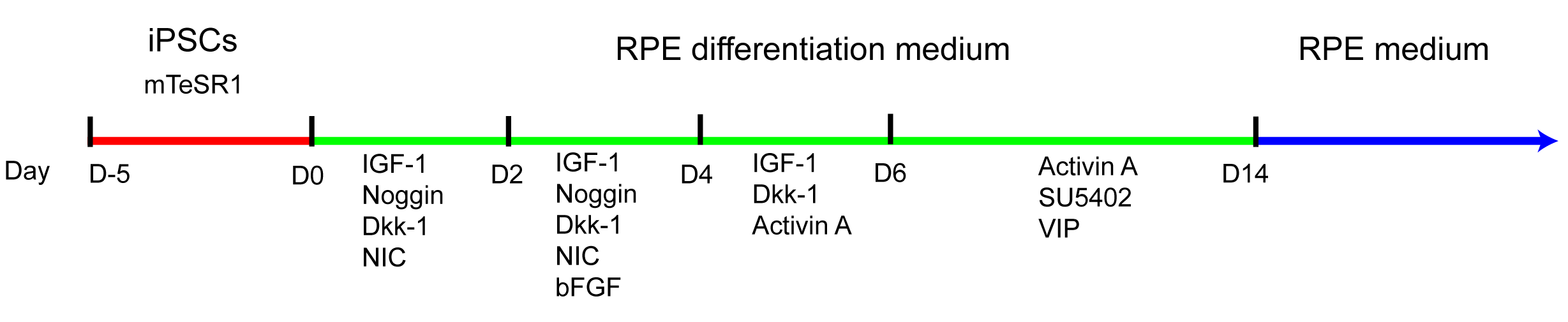

Supplement: Supplementary file 1 — Additional file 1: Fig. S1 hiPSC-RPE differentiation protocol. [file 13287_2020_1608_MOESM1_ESM.tif]

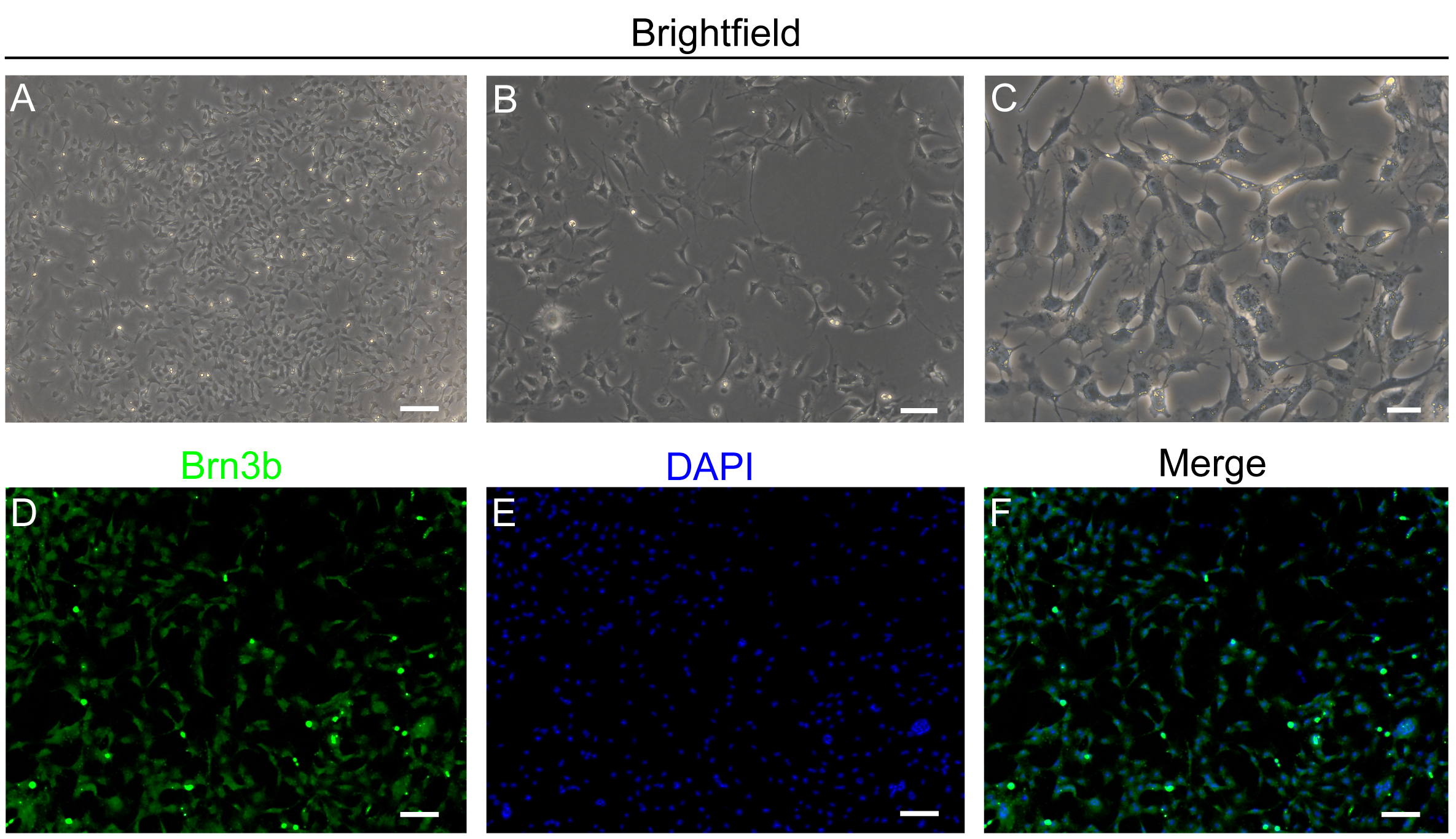

Supplement: Supplementary file 2 — Additional file 2: Fig. S2 Characterization of RGC5. a-c The morphology of RGC5 cell line. d-f RGC-specific marker Brn3b expression in RGC5. Scale bar 200 μm (a), 100 μm (b, d, e, f) and 50 μm (c). [file 13287_2020_1608_MOESM2_ESM.tif]

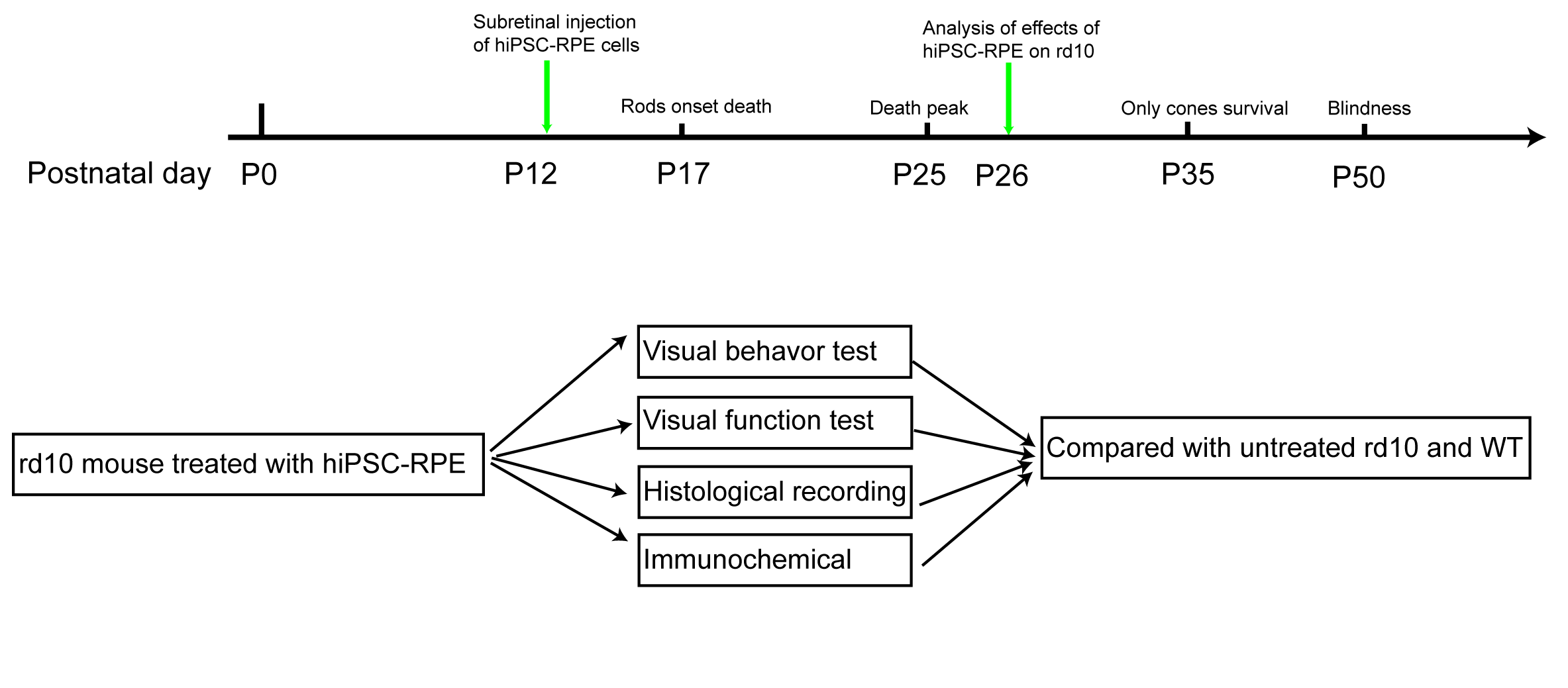

Supplement: Supplementary file 3 — Additional file 3: Fig. S3 Flow chart of the experimental protocol of the animal study. [file 13287_2020_1608_MOESM3_ESM.tif]

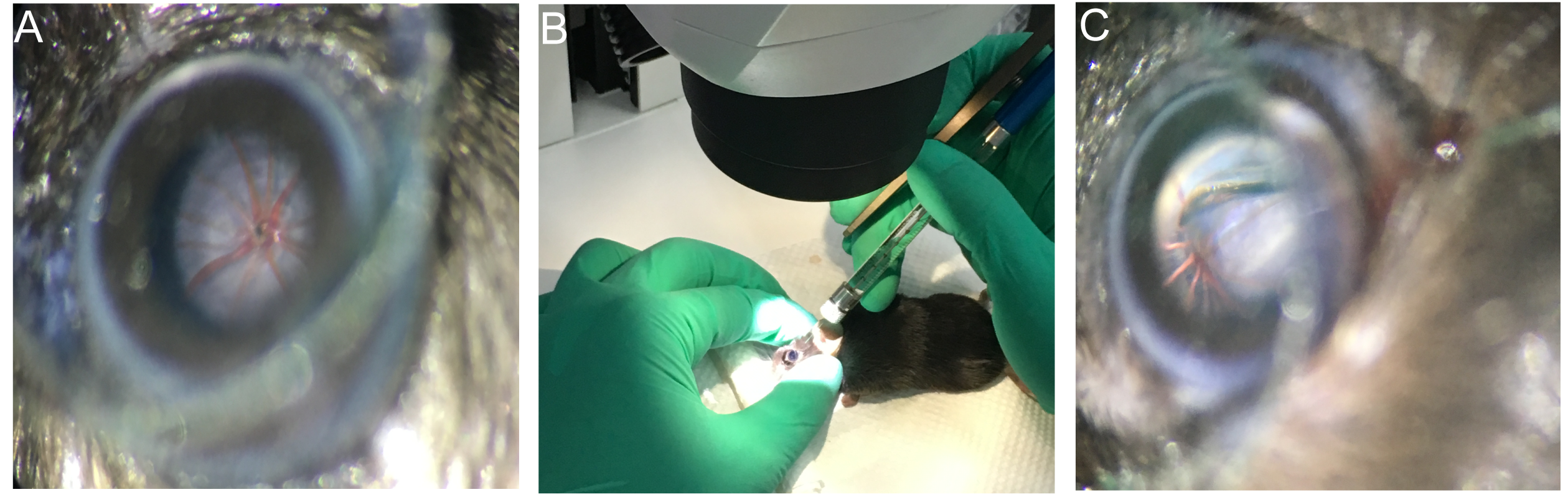

Supplement: Supplementary file 4 — Additional file 4: Fig. S4 Steps of subretinal transplantation in mice. a Dilated pupil before transplantation. b Cells delivery by a 33-gauge needle. c Blebs appeared in the retina after transplantation, the needle bypasses the lens, and the iris to reach the subretinal space followed by injection with 1 μl hiPSC-RPE cells. [file 13287_2020_1608_MOESM4_ESM.tif]

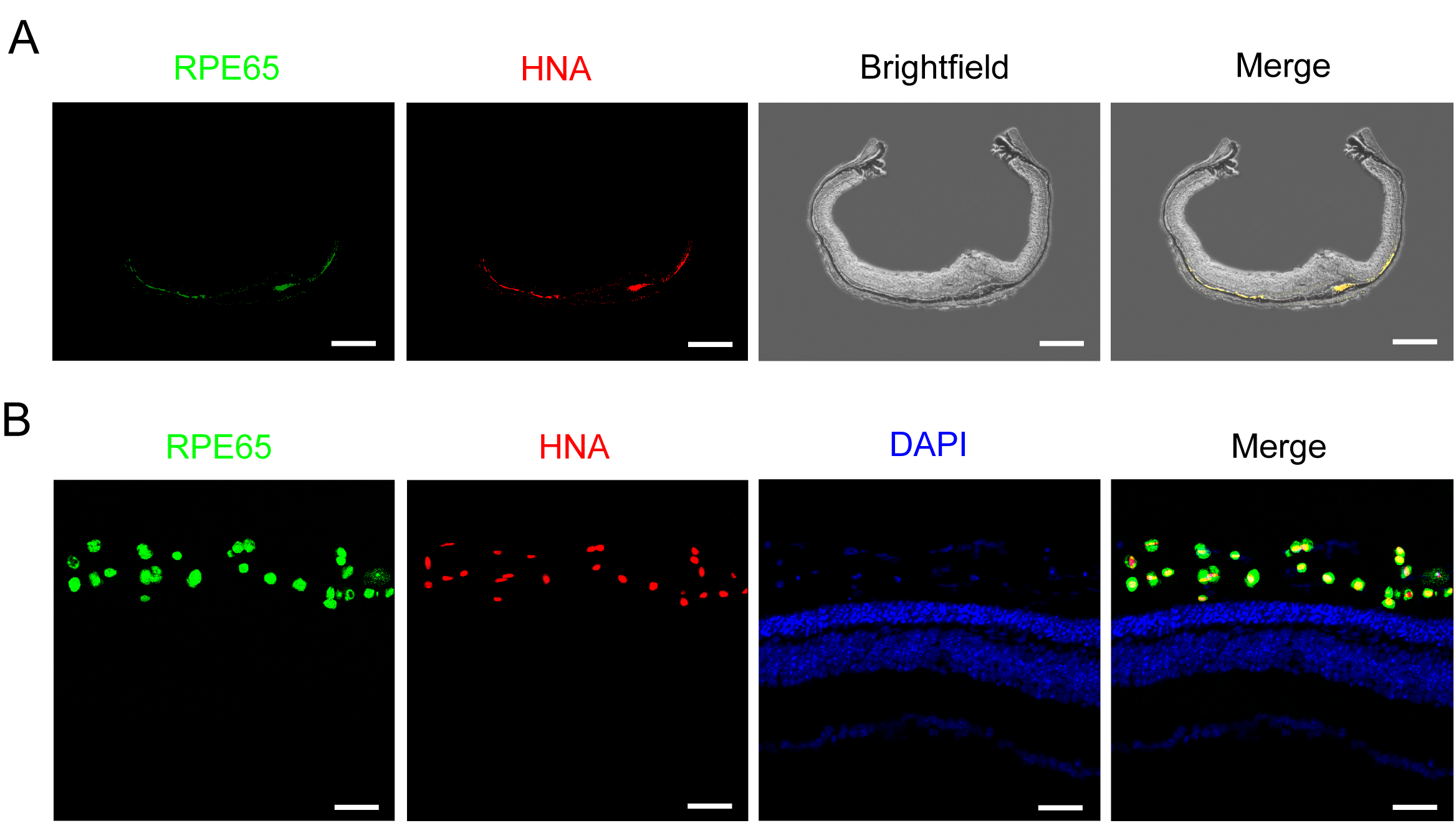

Supplement: Supplementary file 5 — Additional file 5: Fig. S5 hiPSC-RPE cells maintained RPE cell marker RPE65 following subretinal transplantation of rd10. a Histological staining with RPE65 (green) and human nuclear marker (HNA) (red) of cross-sections were obtained from eyes at 2 weeks post-injection of hiPSC-RPE. b Transplanted hiPSC-RPE cells were co-stained with RPE65 (green) and HNA (red), DAPI (blue) stained nuclei. Scale bar 200 μm (a) and 100 μm (b). [file 13287_2020_1608_MOESM5_ESM.tif]
